# Integrated co-expression network analysis uncovers novel tissue-specific genes in major depressive disorder and bipolar disorder

**DOI:** 10.3389/fpsyt.2022.980315

**Published:** 2022-08-23

**Authors:** Mengyao Han, Liyun Yuan, Yuwei Huang, Guiying Wang, Changsheng Du, Qingzhong Wang, Guoqing Zhang

**Affiliations:** ^1^Key Laboratory of Spine and Spinal Cord Injury Repair and Regeneration of Ministry of Education, Orthopaedic Department of Tongji Hospital, School of Life Sciences and Technology, Tongji University, Shanghai, China; ^2^CAS Key Laboratory of Computational Biology, Bio-Med Big Data Center, Shanghai Institute of Nutrition and Health, University of Chinese Academy of Sciences, Chinese Academy of Sciences, Shanghai, China; ^3^Shanghai Key Laboratory of Signaling and Disease Research, Clinical and Translational Research Center of Shanghai First Maternity and Infant Hospital, Frontier Science Center for Stem Cell Research, National Stem Cell Translational Resource Center, School of Life Sciences and Technology, Tongji University, Shanghai, China; ^4^Shanghai Key Laboratory of Compound Chinese Medicines, Institute of Chinese Materia Medica, Shanghai University of Traditional Chinese Medicine, Shanghai, China

**Keywords:** weighted gene co-expression network analysis, integrated analysis, tissue-specific, major depressive disorder, bipolar disorder

## Abstract

Tissue-specific gene expression has been found to be associated with multiple complex diseases including cancer, metabolic disease, aging, etc. However, few studies of brain-tissue-specific gene expression patterns have been reported, especially in psychiatric disorders. In this study, we performed joint analysis on large-scale transcriptome multi-tissue data to investigate tissue-specific expression patterns in major depressive disorder (MDD) and bipolar disorder (BP). We established the strategies of identifying tissues-specific modules, annotated pathways for elucidating biological functions of tissues, and tissue-specific genes based on weighted gene co-expression network analysis (WGCNA) and robust rank aggregation (RRA) with transcriptional profiling data from different human tissues and genome wide association study (GWAS) data, which have been expanded into overlapping tissue-specific modules and genes sharing with MDD and BP. Nine tissue-specific modules were identified and distributed across the four tissues in the MDD and six modules in the BP. In general, the annotated biological functions of differentially expressed genes (DEGs) in blood were mainly involved in MDD and BP progression through immune response, while those in the brain were in neuron and neuroendocrine response. Tissue-specific genes of the prefrontal cortex (PFC) in MDD-, such as *IGFBP2* and *HTR1A*, were involved in disease-related functions, such as response to glucocorticoid, taste transduction, and tissue-specific genes of PFC in BP-, such as *CHRM5* and *LTB4R2*, were involved in neuroactive ligand-receptor interaction. We also found PFC tissue-specific genes including *SST* and *CRHBP* were shared in MDD-BP, *SST* was enriched in neuroactive ligand-receptor interaction, and *CRHBP* shown was related to the regulation of hormone secretion and hormone transport.

## Introduction

With recent advancements in omics study, microarray technology is being increasingly used to uncover the underlying mechanisms of mental illness (1, 2). Major depressive disorder (MDD) and bipolar disorder (BP) are highly complex processes characterized by progressive physiological changes throughout all tissues, it has become increasingly clear that MDD and BP have divergent effects on different tissues at both the gene expression and physiological levels (3–6). In addition, these studies have provided some evidence about the gene expression profiling patterns for specific regions including the cortex, amygdala, and blood, from relatively limited numbers of participants. We should note that transcriptome studies, examining insufficient numbers of participants, are frequently biased toward the identification of high-abundance molecules, producing results that are often difficult to replicate (7). By combining individual microarray studies, integrated analysis can effectively reduce the bias and provide an overall view of gene expression patterns in larger sample sizes.

Meanwhile, the advantage of integrating expression-profiling data according to the source of different materials allows for precise isolation of tissue-enriched or tissue-specific genes or pathways that contributed to mental illness (8). The human brain is an important material for studying psychiatric disorders, carrying out the immense complexity of its precise circuitry, structure, and cellular diversity, and different brain areas have distinct gene expression patterns which manage tissue specificity (9). Although neuroimaging studies have suggested several regional gray matter changes in specified brain areas of MDD or BP, more evidence from different perspectives still needs to be investigated of changes in brain tissue-specific molecular signatures and functions in MDD and BP (10–12). In the current study, we will explore the expression specific characteristics of different areas by means of systematic analysis of larger samples of brain tissues from both diseases.

Major depressive disorder and BP exhibit similar severe depressive symptoms and show less difference in the duration of affective episodes during the course of illness (13). These two illnesses, which share some common phenotypic characteristics and genetic risk factors, are influenced by a combination of multiple genes (14, 15). Additionally, the significant loci susceptible identified from GWAS studies have been found to share genetic risk variants between BP and MDD (16). Another objective of this study is to further determine gene expression patterns and tissue-specific risk factors shared by these two diseases. To clarify the similarities and differences in the tissue-specific expression profiles between these two diseases, we conducted 2 independent analyses [MDD dataset (MDD-), BP dataset (BP-)], and 1 integrated analysis on both MDD and BP dataset (MDD-BP), respectively.

## Materials and methods

### Collection of transcriptional profiling data

**FIGURE 1:**
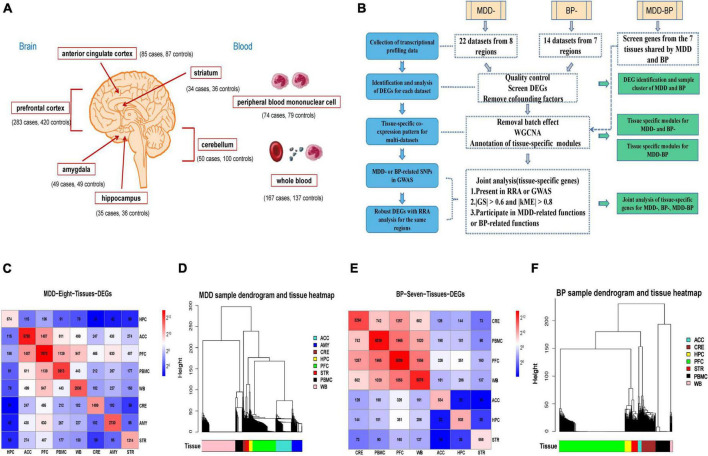
**(A)** Schematic diagram of tissue source and sample quantity. **(B)** Technology roadmap. **(C,D)** Are intersections of the tissues and data were transformed using Log2 as well as hierarchical clustering between different tissue samples in MDD. **(E,F)** Are intersections of the tissues and data were transformed using Log2 as well as hierarchical clustering between different tissue samples in BP.

All the transcriptomic datasets by the platform of mRNA microarray expression profiling on MDD or BP were retrieved using a comprehensive query of Gene Expression Omnibus (GEO). We screened 36 datasets by the following conditions: (1) The datasets included both healthy participants and patients; (2) the datasets containing other factors such as smoking and medication were removed. All demographic information of each participant, which contained age, sex, etc., was extracted from the selected datasets (Figure 1A and Supplementary Table 1). Samples used to obtain mRNA expression-profiling data originated from blood and brain tissues, including the anterior cingulate cortex (ACC), amygdala (AMY), cerebellum (CRE), hippocampus (HPC), prefrontal cortex (PFC), striatum (STR), peripheral blood mononuclear cell (PBMC), and whole blood (WB).

### Identification and analysis of differentially expressed genes from the individual dataset

According to annotation information available on the platform, the probes were converted into corresponding gene symbols, and the raw gene expression values of each gene in different samples were retrieved as a data matrix, which was then normalized with the approach of base-two log transformation. The R *Limma* package (v.3.36) was used to process microarray data for an individual dataset, the differences between patients with MDD or BP and the control group were assessed using a multivariate linear model (17). We selected DEGs with a *p*-value of < 0.05 to screen tissue-specific genes and modules of MDD-, BP-, and MDD-BP from a larger range of DEGs (with a relatively loose threshold). DEGs were then analyzed for subsequent integration analysis of the entire datasets and construction of co-expression networks. Meanwhile, to exclude the false positive possibility caused by confounding factors including age, sex, PH, etc., a *t*-test analysis between gene expression and demographic status of individual participants was performed, and confounding factors genes were removed while looking for tissue-specific genes (Figure 1B and Supplementary Table 2).

### Tissue-specific co-expression pattern by weighted gene co-expression network analysis

#### Removal of batch effect and integration of datasets

The differentially expressed gene expression values were extracted from each dataset and merged into one data matrix according to the same tissue type of two comparisons including MDD vs. control and BP vs. control, thus the 8 data frames from different types of tissues (*n* = 8) have been produced. One study has reported that *ComBat* is superior to other methods in terms of precision, accuracy, and overall performance (18). Then, we applied the *Combat* function in the *sva* package to remove batch effects caused by different technician repetitions for the same material (19). This package based on the empirical Bayes model is widely used to identify, estimate, and remove the mutations generated in experiments from library construction and DNA hybridization to eliminate batch effects. After batch effect calibration, all the collaborated expression values from different tissues for the MDD or BP patients were combined into a final matrix genes (each row was a gene, each column was a sample, and each unit matrix was a sample of mRNA expression levels of a specific gene), which were directly used for subsequent tissue specific co-expression network analysis.

#### Weighted gene co-expression network analysis

Weighted gene co-expression network analysis was used to construct gene co-expression networks for large scale gene expression profiling from various tissues (20). Constructing a weighted gene network entails the choice of the soft-thresholding power β (optimal parameter) to which co-expression similarity is raised to calculate adjacency. We chose a set of soft-thresholding powers ranging from 1 to 20, using the *pickSoftThreshold* function. We calculated the scale-free topological fitting index for several powers and emphasized strong gene-gene correlations at the cost of weak correlations (MDD- soft-threshold power = 7, BP- soft-threshold power = 1, and MDD-BP soft-threshold power = 14), thereby providing appropriate soft-threshold power for network construction (Supplementary Figures 1A,D). Converting adjacency to topological overlap can measure the network connectivity of a gene, defined as the sum of the degree of adjacency between all mutual genes in network generation. Based on Topological Overlap Matrix (TOM) dissimilarities, a hierarchical clustering function was used to group genes with similar expression profiles into modules. Then, we merged the modules with similar expression profiles (Supplementary Figures 1B,C,E,F). In the process of MDD-BP analysis, topological overlap matrices of different datasets may have different statistical properties, so we illustrate a simple scaling that mitigates the effect of different statistical properties to some degree. We scale the MDD TOM as the same 98th percentile as the BP TOM. Meanwhile, based on the same set of DEGs, we performed WGCNA on MDD- and BP-, respectively, and then obtained the same gene modules with different correlation coefficients and *p*-values for MDD- and BP-. Next, we merged the above two results into one based on the following standards. If a module exhibits the same positive or negative in both of the above two independent analyses, it would be regarded as a consistent module, and the smaller value of the two correlation coefficients mentioned above would be selected as the combined value. Otherwise, if a module exhibits no consistency, it will be regarded as an inconsistent module with all parameters of N/A. This process had been demonstrated to study co-expression data of different genders in the handbook of WGCNA (horvath.genetics.ucla.edu/html/CoexpressionNetwork/ Rpackages/WGCNA/Tutorials/, II. Consensus analysis of female and male liver expression data).

#### Annotation of tissue associated modules

The genes in a co-expression module possess a high degree of connectivity. In order to identify the module specified with tissues, an association analysis between tissues and modules was conducted. The function of tissue-specific modules is annotated mainly using the relationship and function of genes in a module, illustrated using the following two aspects. In a module, we selected genes with higher connectivity using the two parameters GS and kME. The parameter kME represents the correlation between the expression of the gene and the first principal component of the module; GS reflects the correlation between gene expression and tissue. We set | GS| > 0.6 and | kME| > 0.8 to screen genes in tissue-specific modules (Supplementary Figures 2, 3 and Supplementary Table 3). Second, the package of *clusterProfiler* was used to evaluate the biological pathways and processes of tissue-specific modules related to diseases (21). After inputting gene IDs that were positively or negatively related to the disease, we performed gene ontology (GO) enrichment analysis examining biological components, molecular functions, biological processes, and KEGG pathways (22, 23).

### Acquisition of robust differentially expressed genes with robust rank aggregation analysis

To measure the robust characteristic of DEGs from multiple datasets, we performed RRA analysis of the datasets from the same tissues of MDD or BP patients (24). Based on assumption that each gene is randomly and freely arranged in each dataset, the rank vector is scored using order-based statistical analysis, and the final score of each vector is designated as the minimum *p*-value. After values were corrected, we determined whether the ranking of a particular gene reached statistical significance, and genes with a *p*-value of < 0.05 were considered significant robust ranking.

### Major depressive disorder- or bipolar disorder-related SNPs in genome wide association study

For all genome-wide single nucleotide polymorphisms (SNPs) significantly associated with MDD or BP, a comprehensive query was made using the GWAS Catalog Database of NHGRI-EBI, which contains GWAS analysis documents and SNP loci. Studies from which variants were collected are determined according to the following conditions: (1) Studies must focus on either MDD or BP; (2) Studies that have indirect phenotypic characteristics, such as obesity and education level, were excluded (25). Finally, 36 GWAS studies on MDD and 51 studies on BP were included and listed (Supplementary Table 4). Raw data were preprocessed and subjected to including merging of recurring risk variants and annotation using ANNOVAR based on GRCh38 assembly (The windows were –20 to 20 Kbp to the loci) (26). We combined the genes obtained using RRA and GWAS and genes in tissue-specific modules. The genes | GS| > 0.6 and | kME| > 0.8 in tissue-specific modules, also present in RRA or GWAS, were regarded as tissue-specific genes.

## Results

### Differentially expressed gene identification and sample cluster of major depressive disorder and bipolar disorder

A total of 22 datasets covering 1,023 participants with MDD and 14 datasets covering 697 participants with BP were downloaded from the repository, and we established strategies for identifying tissue-specific modules, annotated pathways to elucidate tissue biological function, and tissue-specific genes through integrated analysis of those datasets (Figures 1A,B). Here, we focused on the DEGs and their distribution across eight tissues in MDD- and seven tissues in BP- (There was no dataset for AMY in BP). For each dataset, we conducted confounding factor processing (Supplementary Table 2). In the process of multi-datasets integration, we compared the distribution of corrected values before and after the removal to examine the effectiveness of batch effect removal during combining the same tissue datasets. The boxplot showed a more consistent expression level across samples after batch effect performance, the treated batch effect is consistent, which has a distinct distribution of raw untreated datasets (Supplementary Figure 4).

The number of DEGs in different tissues for the MDD- or BP- are shown in Figures 1C,E, respectively. Among the different tissues in the MDD group, PFC tissues contained the highest DEGs (*n* = 7,678), while HPC tissues contained the lowest (*n* = 674). The two brain areas PFC and ACC shared the highest intersection of DEG numbers in different tissues (*n* = 1,487), followed by that between PFC and PBMC (*n* = 1,139). For BP, PFC tissues contained the highest DEG numbers (*n* = 8,089), while HPC contained the lowest (*n* = 938). PFC and WB showed the highest intersection of DEGs (*n* = 1,856), followed by those in PFC and PBMC (*n* = 1,965).

Then, expression matrices of these identified DEGs were extracted from different tissues of patients (only used case samples) with MDD- and BP-, including 481 and 295 samples, respectively (Figures 1D,F). The hierarchical clustering analysis across different tissues has shown that the brain area HPC and STR have the shortest distance both in MDD and BP, while the brain tissues and blood have the maximum distance. Meanwhile, the tissues of PFC, ACC, HPC, and AMY from brain tissues have the closest distance in the hierarchical dendrogram. Thus, we speculated that the similarities or differences of expression patterns across tissues almost reflect to be consistent with the physiological functions and anatomical locations among these tissues.

### Determination of tissue specific modules for major depressive disorder and bipolar disorder individually

A total of 11,997 DEGs from the MDD tissues and 11,025 DEGs from BP tissues were combined into an overall expression matrix as input data. Using WGCNA, 17 and 18 co-expression patterns (modules) were assigned to the MDD- and BP- datasets, respectively. Correlations between each module and each tissue were evaluated to identify tissue-specific modules, for which the threshold was set as an absolute value of correlation coefficient | r| > 0.6 and *p*-value < 0.05.

In MDD-, nine tissue-specific modules across different tissues were identified and distributed across the 4 tissues, including ACC [darkslateblue module (*r* = 0.6)], CRE [lavenderblush3 module (*r* = 0.9), and brown4 module (*r* = –0.83)]; PFC [bisque4 module (*r* = 0.8) and lightcoral module (*r* = –0.94)]; PBMC [lightskyblue module (*r* = 0.85) and darkolivegreen module (*r* = –0.96)]; and WB [lightgreen module (*r* = 0.98) and saddlebrown module (*r* = –0.91)]. For BP-, six tissue-specific modules were found in the tissues: CRE [darkgreen module (*r* = 0.92) and greenyellow module (*r* = –0.95)], PFC (pink module (*r* = 0.93) and brown module (*r* = –0.94)], and PBMC [yellow module (*r* = 0.83) and blue module (*r* = –0.98)].

**FIGURE 2:**
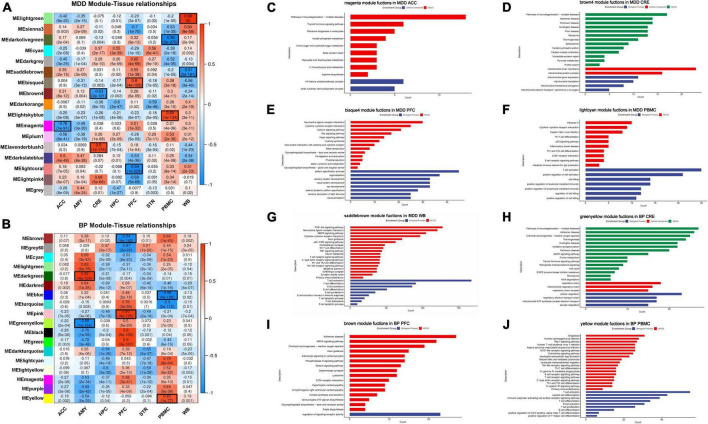
**(A,B)** Correlation and *p*-value between co-expression network modules and tissues in MDD- and BP-. Correlation coefficient and *p*-value are shown, and we defined the absolute correlation coefficient > 0.6 and *p*-value < 0.05 as the tissue-specific module. **(C–J)** GO and KEGG analysis results of certain tissue-specific modules. We focus on the tissue-specific module, the results of functional enrichment analysis showed that ACC is related to histone modification, CRE is participant in mitochondrial and ribosome function, PFC is related to cognition, and blood tissue have a primary role in immune function.

Gene ontology and KEGG pathway analysis were conducted to annotate the biological functions of these tissue-specific modules (Supplementary Table 5). We speculated that these tissue-specific modules have tissue-specific biological functions, some functions have been widely reported in the literature, and we illustrated this in the results (Figure 2). From the GO results of MDD, the biological function of ACC associated modules is mainly related to a histone modification and the enriched GO terms are involved in the formation of the H4 Histone acetyltransferase complex; it has been suggested that abnormal activation of histone modification and epigenetic inhibitory system may be related to the pathogenesis of MDD in ACC (27). In CRE tissues, the top significantly enriched GO functions are mainly associated with mitochondrial membrane formation and mitochondrial cytochrome transport, while CRE in BP is associated with respiratory chains, such as mitochondrial respiration and cellular respiration. Our view is also supported by a meta-analysis showing that the loss of mitochondrial electron transport chain complex in CRE has an effect on MDD and BP (28). Interestingly, the epigenes of PFC-associated modules are significantly enriched perception-related functions, which are manifested in the conduction of vision and taste, PFC in BP is associated with receptor activity. Overall, blood pathways mainly belonged to the immune-inflammatory imbalance and kynurenine pathway, while brain tissue pathways were enriched in the hypothalamic-pituitary-adrenal axis (HPA), circular rhythm abnormalities, mitochondrial dysfunction and oxidative stress, and changes in neuroplasticity and neurotrophic signaling. These ideas are conforming to the accepted hypothesis that falls within mood disorders (29–31).

Apart from these tissue-specific modules, we also found that some tissue-unspecified modules have a high correlation with two different tissues simultaneously, implying to share the same biological functions. The cyan and dark orange module correlated with both HPC and STR of MDD, and mainly enriched in metabolism pathway and o-glycan biosynthesis, respectively. Similarly, the dark turquoise and light yellow module also have a higher correlation with both HPC and STR of BP, and the pathway analysis was related to nutrition-related pathways. Interestingly, HPC and STR also have closer distance and similarities in the above-mentioned hierarchical clustering. The relationship between HPC and STR has also been reported; HPC and STR are both related to temporal processing of memory; the association between real-world experiential diversity and positive affect relates to hippocampal-striatal functional connectivity (32, 33).

### Functional analysis of co-expression in major depressive disorder bipolar disorder

**FIGURE 3:**
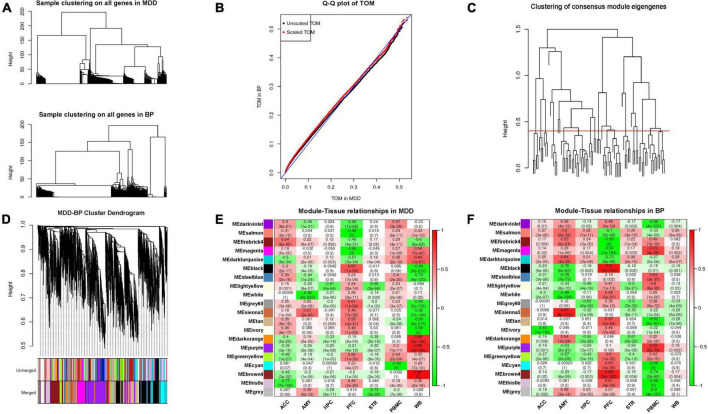
**(A)** Hierarchical clustering using shared genes is similar to the clustering used for single disease. **(B)** Quantile-quantile plot of TOMs in MDD and BP. The black points are TOMs before scaling, and the red points are TOMs after scaling. The closer the points lie to the reference line shown in blue, the closer the distribution of TOM values in the two datasets. The closer the points in the graph are to the reference line marked in blue, the more similar the TOM distributions of the two datasets are. **(C)** The hierarchical clustering graph between co-expression network modules. Groups of eigengenes below the threshold represent modules whose expression profiles are too similar and should be merged. **(D)** Gene dendrogram obtained by clustering the dissimilarity based on consensus topological overlap. The two-color rows show the preliminary (unmerged) and final (merged) module assignments; in this study, 20 modules were obtained. **(E,F)** MDD and BP mainly contain modules with consistent expression and inconsistent expression.

**FIGURE 4:**
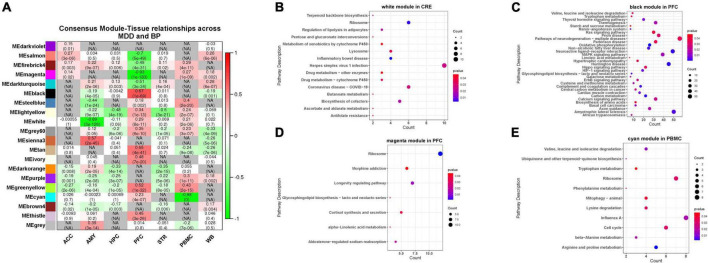
**(A)** Correlation between MDD-BP co-expression network and tissue. Here, the smaller absolute correlation coefficients and *p*-value between MDD- and BP- was shown, while N/A represents for inconsistent modules. **(B–E)** We focus on the modules with the consistency of positive and negative correlations in the two diseases, here we showed functional analysis of consistent modules, including CRE (white modules), PFC (black and magenta modules), and PBMC (cyan module).

We screened a total of 7,987 genes (intersection of MDD- and BP- DEGs) from the 7 tissue samples shared by MDD and BP (except AMY) and constructed a co-expression network module based on the matrix of these genes. After parameter optimization, it is ensured that the characteristic genes of each module have the same distribution in MDD-BP. The two diseases of BP and MDD are analyzed for the correlation of the organization, and then the results of the two correlations analysis are merged. We focus on the modules with the consistency of positive and negative correlations in the two diseases and set N/A for the inconsistent modules (Figures 3A–F, 4A).

We focused on the consistency module of MDD-BP and observed that the white module in CRE, the black and magenta modules in PFC, and the cyan module in PBMC showed similar positive or negative tendencies. These results suggest that the KEGG pathway of the black module in PFC (*r* = 0.67) was mainly related to metabolism and neuronal processes, such as carbon metabolism and neuroactive ligand-receptor interaction, while the magenta module in PFC (*r* = –0.93) was mainly involved in cortipair synthesis and secretion, glycosphingolipid biosynthesis-lacto and neolacto series, and morphine addiction. The cyan module in PBMC (*r* = –0.98) was correlated with amino-acid metabolism-related pathways (Figure 4).

### Joint analysis of tissue-specific genes in different tissues

After GWAS systematic analysis, we obtained 678 and 790 disease-related genes, respectively (Supplementary Table 4). The robust DEGs for RRA were sorted according to tissue logFC, and the number of genes ranged from 148 to 469 in each tissue type (Supplementary Table 6). Pearson correlation analysis was further conducted with tissue-related gene expression and demographic information of participants to exclude the effect of confounding factors. These disease-related genes from GWAS and RRA were verified carefully and were marked with participating functions to determine tissue-specific disease-related genes in MDD- or BP- group.

In MDD, we identified 163 tissue-specific disease-related genes that participated in the function of the ACC, CRE, PFC, PBMC, and WB. In BP, we identified 122 tissue-specific disease-related genes in CRE, PFC, and PBMC, and 16 tissue-specific genes in both MDD and BP from CRE, PFC, and PBMC (Supplementary Table 7). Tissue-specific genes were involved in several important biological processes related to MDD and BP.

In MDD-, as the tissue-specific genes of the bisque4 module in PFC, *IGFBP2* is mainly involved in response to glucocorticoids and corticosteroids, while *HTR1A* participated in an important sensory pathways: taste transduction. For PBMC, the tissue-specific gene of the light skyblue module is *TNFSF4*, which jointly plays a critical role in immune response and regulatory T cell differentiation. For WB, tissue-specific genes in the saddle brown module participate in the immune-related pathway. In BP-, the pink module or PFC, *CHRM5, and LTB4R2* were the tissue-specific genes enriched in neuroactive ligand-receptor interaction and calcium signaling pathway. In the brown module, the tissue-specific genes *PIK3R1* and *WNT5A* participated in the axon guidance and Alzheimer’s disease. Tissue-specific genes of the PBMC yellow module participated in the immune-related pathway.

**FIGURE 5:**
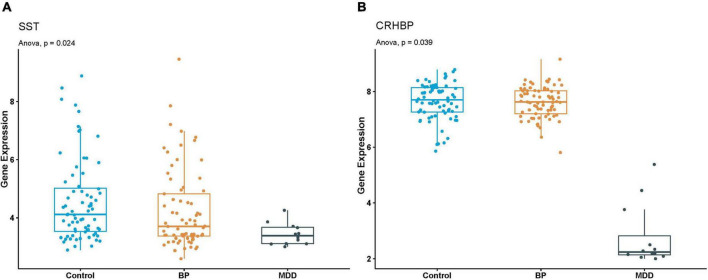
**(A,B)** Boxplot of *SST* and *CRHBP* expression in GSE87610. *P*-value of Anova analysis were also presented for each gene in Control, BP and MDD samples.

In MDD-BP, we found the tissue-specific gene *SST* and *CRHBP* in the black module of PFC in MDD and BP. The *SST* gene was enriched in neuroactive ligand-receptor interaction, and the tissue-specific gene *CRHBP* showed is related to the regulation of hormone secretion and hormone transport. To further validate the expression of *SST* and *CRHBP* genes in MDD and BP, another dataset (GSE87610) was collected using PFC obtained from patients with MDD and BP and healthy controls. As shown in Figure 5, the mRNA levels of *SST* and *CRHBP* were significantly downregulated in PFC obtained from patients with MDD and BP. Based on the results of the WGCNA analysis, it can be concluded that *SST* and *CRHBP* may be candidate marker genes of MDD and BP.

## Discussion

Tissue-specific genes are a class of genes that are highly expressed in specific tissues and play a role in transcriptional regulation, development, stress response, and even disease development (34–36). Therefore, the study of brain-specific genes is conducive to comprehensively understanding the function and mechanism of mood disorder. Since Genotype-Tissue Expression (GTEx) project has completed RNA sequencing data of 1,641 samples across 43 tissues from 175 individuals and some tissue-specific genes, gene networks and genetic variants have been identified to address each respective tissue’s unique functions (37). A study described the methods for analyzing numerical approximations of tissue specificity of human genes to identify candidate cancer biomarkers. The results show that most human genes (nearly 98%) are expressed with low specificity in many tissues, and only a few genes have very specific tissue expression profiles. These tissue-specific genes are important for selecting new therapeutic targets and new diagnostic serum biomarkers (38). Another study developed a computational pipeline for calculating the prioritization of disease-gene candidates by integrating tissue-specific, gene expression, protein-protein interaction networks, ontology-based similarity, and topological measures. By means of this pipeline, a list of 32 prioritized Alzheimer’s disease (AD) genes were produced and two AD susceptibility genes including PSEN1 and TRAF1 were correctly identified (39). However, it is still very challenging to determine the tissue-specific functions of various brain regions, although the recent emergence of spatial transcriptomic has provided the possibility to study the tissue specificity of brain regions (40). To the best of our knowledge, this study is the first to report on overall functional similarities and specificities between tissues obtained from patients with MDD and BP.

Weighted gene co-expression network analysis is essential to elucidate co-expression patterns or hub genes from genome-wide expression profiling (41, 42). Most WGCNA studies focused on identifying the modules associated with certain phenotypes and extracting the potential hub genes centered as co-expression networks on the basis of connectivity. In the current study, we utilized the algorithms of WGCNA and explored the association between tissue and co-expression patterns to identify the tissue-specific genes and pathways. Single dataset analysis often makes the results difficult to repeat due to the characteristics and errors of the data set itself (7), and it is more to show the characteristics of its own single data set. Similarly, there are risks in the data integration process, and bias may come from removing batch effects from multiple datasets or reducing the characteristics of a single set of data. However, in general, the use of multi-data combined analysis is more in line with the purpose of our research, which can more comprehensively understand the overall occurrence and development of the disease, and screen more credible and reliable disease functional modules.

We emphasized the need to focus on tissue-specific modules, the brain tissue-specific modules were analyzed to be at a significant level for the association between co-expression genes and tissues, which also reflect the biological functions of the differential genes specifically enriched into a particular tissue of patients who are depressed. In the study of MDD and BP, blood and brain tissue samples are always the main sample sources. We have included both kinds of tissues in our research to compare their similar yet different aspects in the development of diseases from an overall perspective. Our results showed that MDD and BP blood samples contained several tissue-specific modules, including epigenes enriched in immune and inflammatory pathways, such as the Th1 and Th2 cell differentiation pathway and human T-cell leukemia virus 1 infection pathway. Immune and inflammatory pathways have been detected in MDD and BP blood samples using single-disease analysis, indicating that the two diseases are likely caused by an immune disorder affecting the function of the CNS (43–45). Preclinical studies and those examining postmortem brain tissues have shown that cytokine-related inflammatory mechanisms play contributory roles in depression. Additionally, compounds, including infliximab and sirukamab, targeting inflammatory genes show antidepressant effects (46, 47). Our present study also highlights the essential role of inflammation-related immune processes in depression. Interestingly, changes in the expression profile of blood can partially reflect changes in brain tissue (48). In tissue similarity, with respect to physiological function, HPC and STR are both related to temporal processing of memory and hippocampal-striatal functional connectivity. In sum, we presumed that MDD and BP are systematic disorders having tissue-specific abnormalities of the brain and blood.

On the basis of tissue-specific modules, we found 1,074 tissue-specific genes. Some of these genes have been reported previously, while others were newly found in our present study. *IGFBP2*, a tissue-specific gene in PFC tissues, mainly responded to glucocorticoids and corticosteroids, appear to play a governing role in insulin-like growth factor (*IGF*) regulation in the central nervous system. This protein in the alterations in neurodevelopment and neuroprotection has been observed in mood disorder by another study (49). *HTR1A*, which is one of the serotonin receptor polymorphisms *(5-HTR1A, 5-HTR2A*), is suggested to be involved in the pathogenesis of MDD and the antidepressant treatment response (50–52). Tissue-specific gene *CHRM5*, which is one of the receptors in the cholinergic system, and the antidepressant imipramine have a better therapeutic effect (53, 54). Another tissue-specific gene, *LTB4R2*, in which the exon region was identified as the number one depression-related differential methylation region (*P* = 1.27 × 10^–14^) (55). It has been reported that *PIK3R1* is a potential target of BP from the genome and methylation groups, respectively, and a vector machine was adopted to fit different gene combinations to evaluate diagnostic value for bipolar disorder, the combination “*PIK3R1* + *FYN*” in the SVM model showed the best diagnostic value (56–58). Our results indicate that *WNT5A* is a tissue-specific gene in PFC, during neuro-development, and in the adult brain, the Wnt signaling pathway plays a crucial role in neural stem-cell proliferation, differentiation, and migration, and in neuroplasticity and neurogenesis. Wnt signaling is triggered in cell autonomously by time-dependent expression of *WNT5A* and activation of non-canonical signaling (59–61). *ADCY2* is involved in cortisol synthesis, and elevated cortisol levels affect HPA axis activity (30, 31, 62). Therefore, tissue-specific genes may affect the pathogenesis and pathophysiology of MDD and BP.

A recent meta-analysis examining genome-wide studies of eight psychiatric disorders, including MDD and BP, found that genetic factors were shared within these eight psychiatric disorders (63). Psychiatric polygenic risk score (PRS) models, based on genotypic and phenotypic data, can modestly discriminate between MDD and BP (64). Our present study is the first to use WGCNA and joint analysis to comprehensively screen for sharing signature genes at the tissue level. We found that tissue-specific genes *SST* and CRHBP were shared in both MDD and BP; this finding was validated by independent studies on MDD and BP, which are detailed in what follows. Studies examining patients with MDD and human postmortem brain tissues have shown that somatostatin, encoded by *SST*, significantly decreases in the dorsolateral PFC (dlPFC), subgenual anterior cingulate cortex (sgACC), and the AMY (65). Studies in patients with BD also found reduced levels of somatostatin and somatostatin-related neurons in various brain tissues (66). Similar changes were found in patients with schizophrenia and AD (67). These findings suggest that reduced somatostatin expression directly contributes to mood dysfunction. A candidate gene association study has shown that *CRHBP* is involved in modulating treatment using SSRI antidepressants (68). Additionally, decreased *CRHBP* expression has been shown in the tissues of men with MDD, BP, and SCZ (69, 70). Combined validation of independent datasets has shown that dysregulated mRNA expression of *SST* and *CRHBP* is shared in MDD-BP, and this plays a critical role in the etiology of mood disorders. These findings suggest that genes found in our combined analysis can be used to obtain reliable characteristics of gene-expression patterns and pathways.

## Conclusion

In conclusion, our comprehensive integrated analysis of multi transcriptional studies, GWAS, and RRA successfully found tissue-specific gene modules and their function in MDD and BP. Additionally, we investigated the co-expression patterns and identified tissue-specific genes that play important roles in mood disorders.

## Data availability statement

The datasets presented in this study can be found in online repositories. The names of the repository/repositories and accession number(s) can be found in the article/Supplementary material.

## Author contributions

GZ, QW, and CD designed the project. MH and LY performed the WGCNA and integrated analysis. YH provided the view of batch effect removal. MH and LY wrote the first draft of the manuscript. GZ, QW, CD, and GW checked and edited the manuscript. All authors read and approved the final version of the manuscript.
