# Supplementary material for: Integrated co-expression network analysis uncovers novel tissue-specific genes in major depressive disorder and bipolar disorder
Source: Front Psychiatry. 2022 Aug 23;13:980315. doi: 10.3389/fpsyt.2022.980315 (PMC9445988; doi:10.3389/fpsyt.2022.980315)
Supplement: Supplementary file 8 [file Data_Sheet_1.DOCX]

**Supplementary Figures**


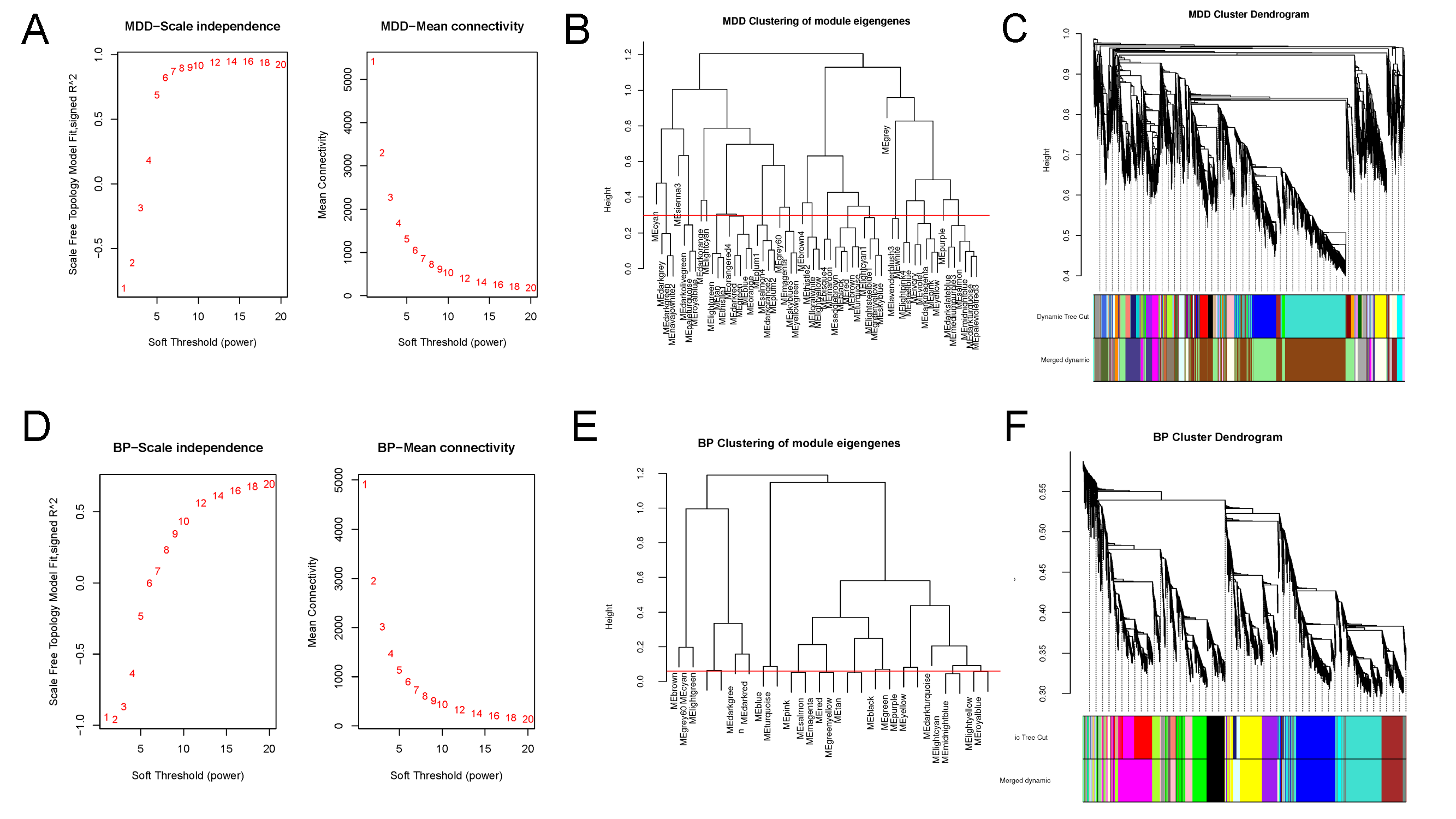


Figure S1. (A) (D) Analysis of network topology for various soft-thresholding powers. Thresholds 7 and 1 were used to construct the co-expression network for MDD and BP, respectively; (B) (E) The hierarchical clustering graph between co-expression network modules. (C) (F) Gene dendrogram obtained by clustering the dissimilarity based on consensus topological overlap. The two-color rows show the preliminary (unmerged) and final (merged) module assignments. The similar modules were combined and resulted in 17 and 18 modules, respectively.


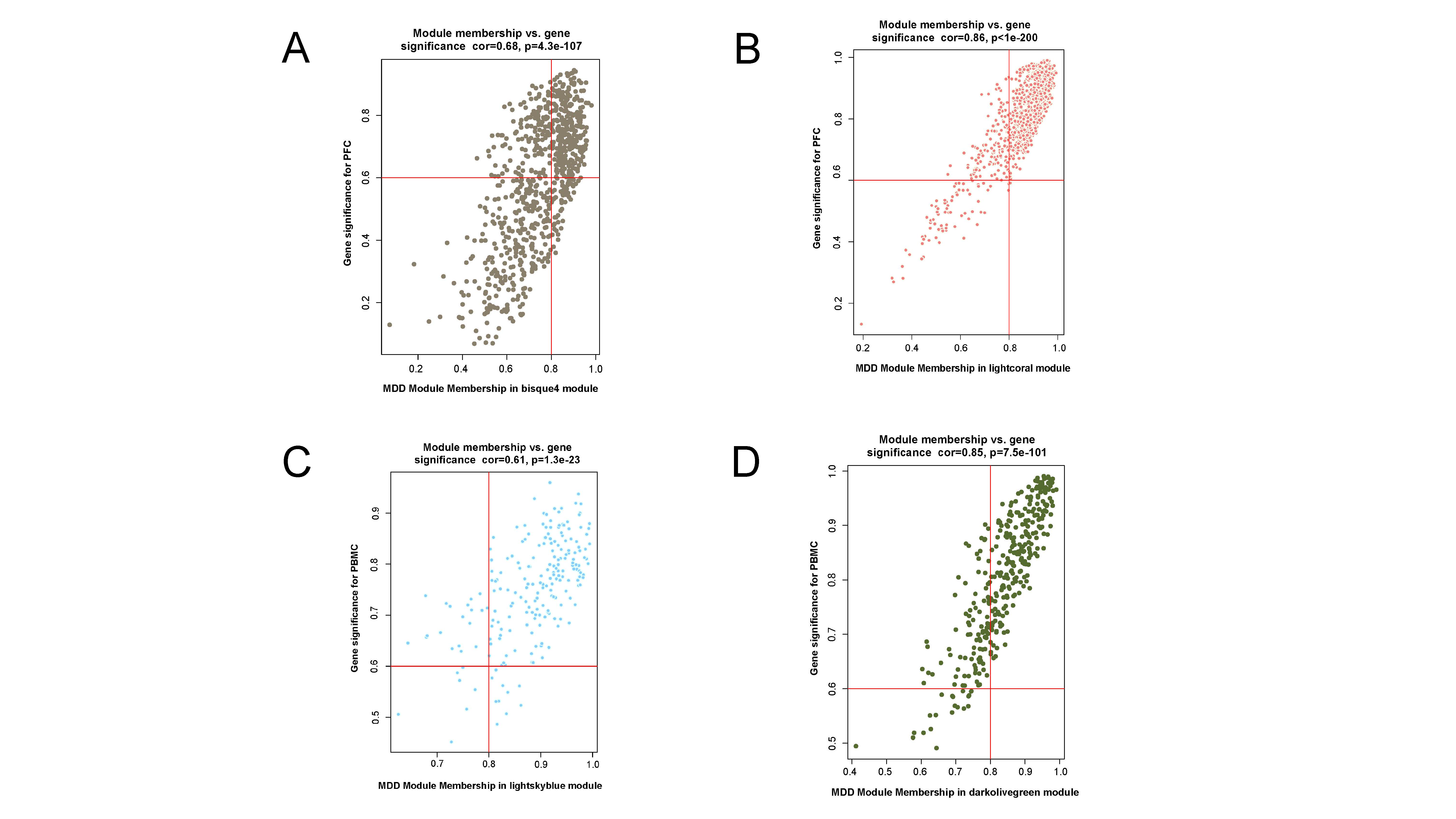


Figure S2. Scatter plots of genes in the tissue-specific modules. The condition |GS| > 0.6 and |kME| > 0.8 to screen genes in tissue-specific of PFC and PBMC in MDD.


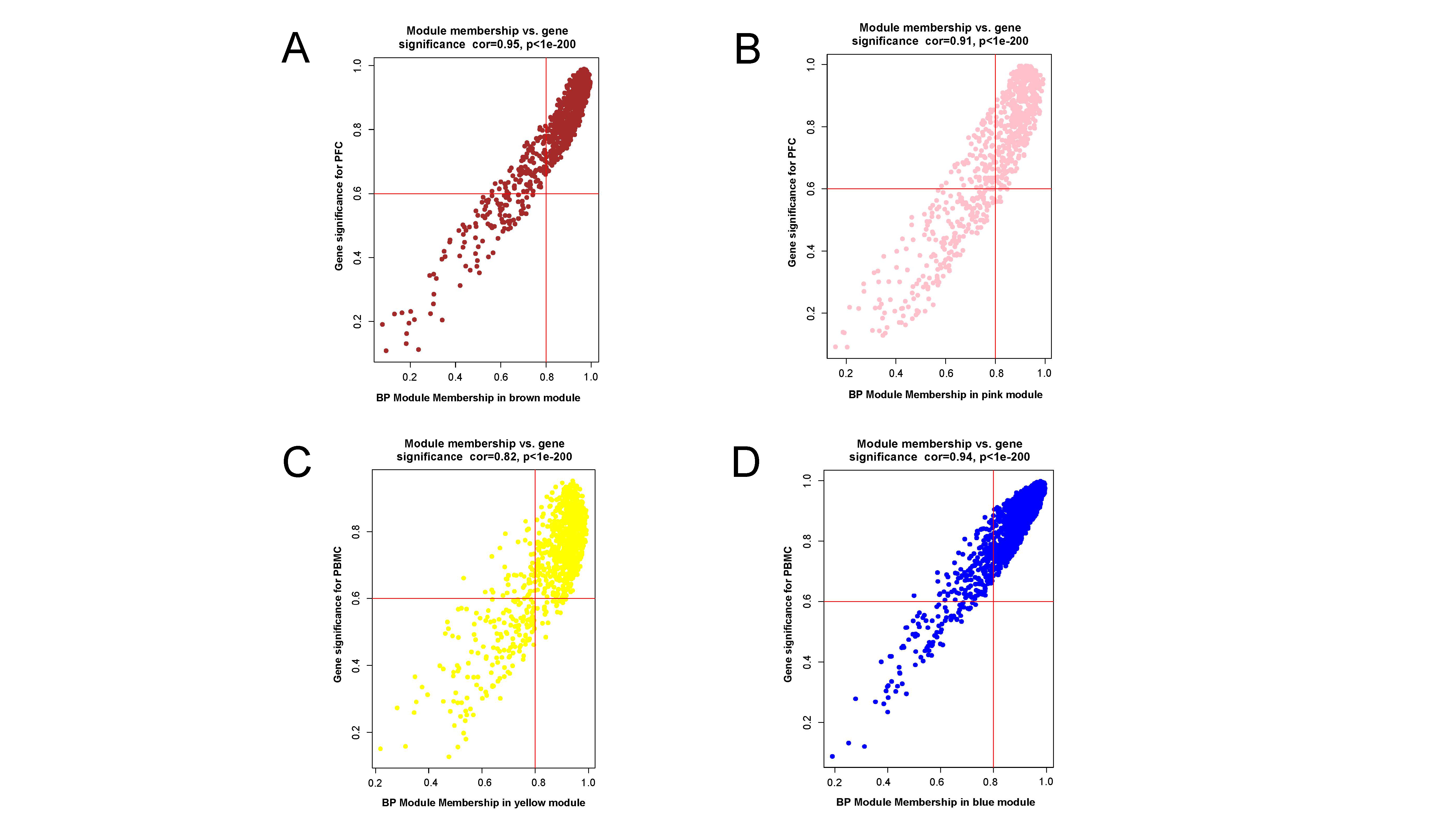


Figure S3. Scatter plots of genes in the tissue-specific modules. The condition |GS| > 0.6 and |kME| > 0.8 to screen genes in tissue-specific of PFC and PBMC in BP.


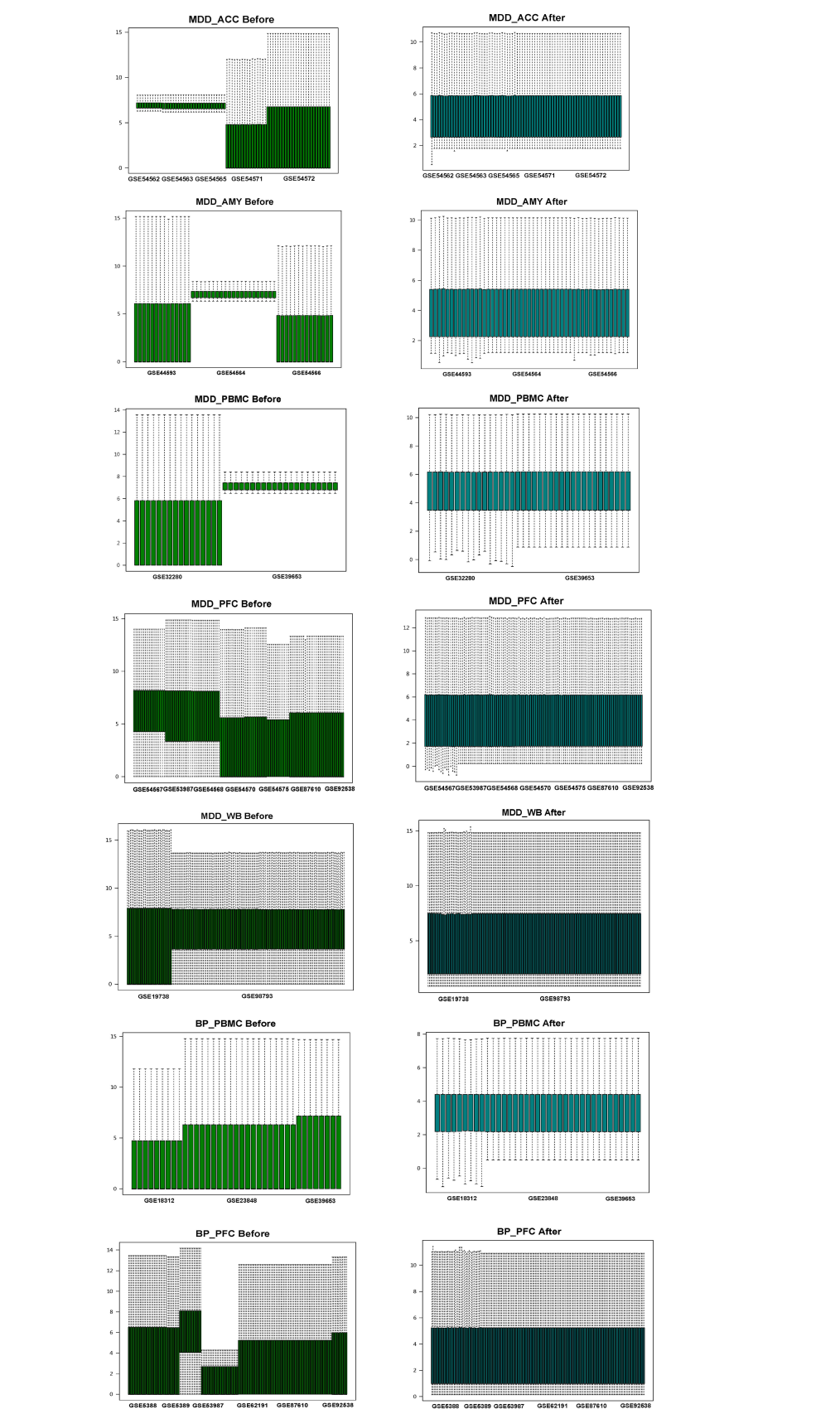


**Expression**

**Before**

**After**

Figure S4. Boxplot of comparing the results before and after batch effect removal for each tissue type. For the same tissue containing multiple data sets, we removed batch effect when data sets were combined. The figure above shows the changes of data expression before and after the removal of batch effect.
